# SNORA47 affects stemness and chemotherapy sensitivity via EBF3/RPL11/c-Myc axis in luminal A breast cancer

**DOI:** 10.1186/s10020-025-01216-3

**Published:** 2025-04-22

**Authors:** Qilin Han, Ying Zhou, Zixian Dong, Weitao Wang, Menghan Wang, Mengyang Pang, Xinyue Song, Bo Chen, Ang Zheng

**Affiliations:** 1 Department of Breast Surgery, the First Hospital of China Medical University, 155 Nanjing North Street, Heping District, Shenyang, Liaoning 110001 China; 2 College of Life Science, China Medical University, Shenyang, China; 3 Department of Pharmacology, School of Pharmacy, China Medical University, Shenyang, China

**Keywords:** Luminal A breast cancer, SNORA47, Chemotherapy sensitivity, Stemness, EBF3

## Abstract

Chemotherapy sensitivity is an important factor that restricts the prognosis of breast cancer, and breast cancer stem cells (BCSCs) are the root cause of chemotherapy sensitivity. SNORA47, a member of the small nucleolar RNAs, has not been documented in the context of breast cancer, although it has been reported in lung cancer. In this study, high SNORA47 expression was linked to unfavorable survival outcomes among patients with Luminal A breast cancer in The Cancer Genome Atlas (TCGA). Among Luminal A patients, an elevated expression of SNORA47 correlated with high TNM stage (*P* = 0.049). SNORA47 was strongly associated with breast cancer stemness phenotype and tumor sensitivity in vivo and in vitro. Our findings demonstrated that SNORA47, through its interaction with early B-cell factor 3(EBF3), facilitated the translocation of ribosomal protein L11(RPL11), which as a modulator that subsequently regulates the expression levels of the oncogene c-Myc. These discoveries provided novel insights into the molecular mechanisms of breast cancer progression and suggested potential therapeutic targets for overcoming drug sensitivity by disrupting the SNORA47-EBF3-RPL11 axis.

**Supplementary Information:**

The online version contains supplementary material available at 10.1186/s10020-025-01216-3.

## Introduction

Breast cancer constitutes one of the most prevalent malignant neoplasms among females globally, thereby posing a grave peril to both physical and psychological well-being (Siegel et al. 2023). It is a highly heterogeneous disease, with significant variability in treatment approaches and prognoses across different molecular subtypes. Among them, the Luminal A subtype, has a relatively favorable prognosis (Lukey et al. 2019). However, approximately 28% of Luminal A tumors exhibit lymph node metastasis at initial diagnosis, which is linked to poorer outcomes (Sawaki et al. 2014). Furthermore, 25% of Luminal A tumors will relapse within the first 5 years, with the risk of recurrence significantly tripling between the 5th and 10th years (Ignatov et al. 2018). Some patients may experience recurrence many years after surgery, with relapsed tumors often becoming more aggressive (Ogba et al. 2014).

Chemotherapy is recommended for some high-risk patients with Luminal A subtype. It is a future treatment idea to screen high-risk patients for chemotherapy sensitivity group for early intervention to reduce relapse. Breast cancer stem cells (BCSCs) are the root cause of chemotherapy sensitivity. A more profound comprehension of the molecular mechanisms that underpin these stemness and resistant phenotypes is imperative for the advancement of more efficacious treatments. Therefore, we aim to investigate biomarkers which indicate stemness and chemotherapy sensitivity in Luminal A breast cancer patients.

Small nucleolar RNAs (snoRNAs) have emerged as significant regulators in cancer biology, influencing various cellular processes, such as ribosome biogenesis, RNA modification, and gene expression regulation (Esteller 2011; Bratkovič and Rogelj 2014). SnoRNAs are RNAs with conserved secondary structure and no protein coding function in the nucleolus, with lengths ranging from 60 to 300 nt (Persson et al. 2020; Liang et al. 2019). SnoRNAs are composed mainly of the box H/ACA snoRA and the box C/D snoRD families (Massenet et al. 2017). The H box (ANANNA) and ACA box conserved sequence elements constitute the box H/ACA snoRA (McCann et al. 2020), which binds to target RNA and catalyzes the conversion of uridine to pseudouridine, thereby enhancing target specificity. On the other hand, box C/D snoRD can bind to ribonucleoproteins to form snoRNPs complexes and participate in the 2’-O-methylation of rRNA (A I L 1989), thereby altering its structure and enhancing its hydrophobicity. In recent years, studies have shown that snoRNAs are strongly associated with breast cancer development (Escuin et al. 2024)and metabolism (Zhang et al. 2023) and even served as biomarkers for breast cancer diagnosis (Li et al. 2024). Notably, changes in the levels of core proteins, such as FBL (fibrillarin), which is a component of box C/D snoRNPs, have been linked to cancer progression by influencing Myc levels and p53 activity (Nicholas et al. 2004). Additionally, snoRNA U50, which belongs to the box H/ACA type, has been implicated in tumor suppression through its role in mediating methylation in 28 S rRNA. These findings suggest that alterations in snoRNA expression and function may play a significant role in the development of breast cancer (T et al. 2013).

Our previous findings revealed that SNORA47 was significantly over expressed in MCF-7 MS cells than in MCF-7 (Shan et al. 2024). SNORA47, which belongs to the box H/ACA snoRA families. SNORA47 was reported to affect lung cancer progression through the regulation of EMT and apoptosis (Yu et al. 2021). Prior research has demonstrated that the upregulation of SNORA47 occurs in non-small cell lung carcinoma and that it plays a role in the PI3K/Akt signaling cascade (Braicu et al. 2019; Li et al. 2017). Nevertheless, there has been no reported literature on the role of SNORA47 in relation to breast carcinoma.

C-Myc was frequently dysregulated in breast cancer, contributing to tumor initiation, progression, and therapeutic resistance (Akimasa et al. 2021). The RPL11/c-Myc pathway was known to impact the expression of downstream genes involved in cancer cell growth and survival (Lee et al. 2023). By RNA pull down, we found that the early B-cell factor 3 (EBF3) might bind SNORA47. It has been reported that EBF3 directly combines with Vimentin promoter and up-regulates its expression, promoting epithelial-mesenchymal transition (EMT) transformation and metastasis of nasopharyngeal carcinoma (Ding et al. 2022a, b, c).

The purpose of this research is to elucidate the intricate molecular interactions among SNORA47, EBF3, and RPL11 in breast cancer cells. These findings suggest that targeting the SNORA47-EBF3/RPL11/c-Myc axis is a useful strategy to influence chemotherapy sensitivity and to ascertain potential therapeutic targets for the enhancement of treatment efficacy in breast cancer.

## Results

### SNORA47 is over-expressed in luminal A breast cancer and indicated poor prognosis

**Fig. 1:**
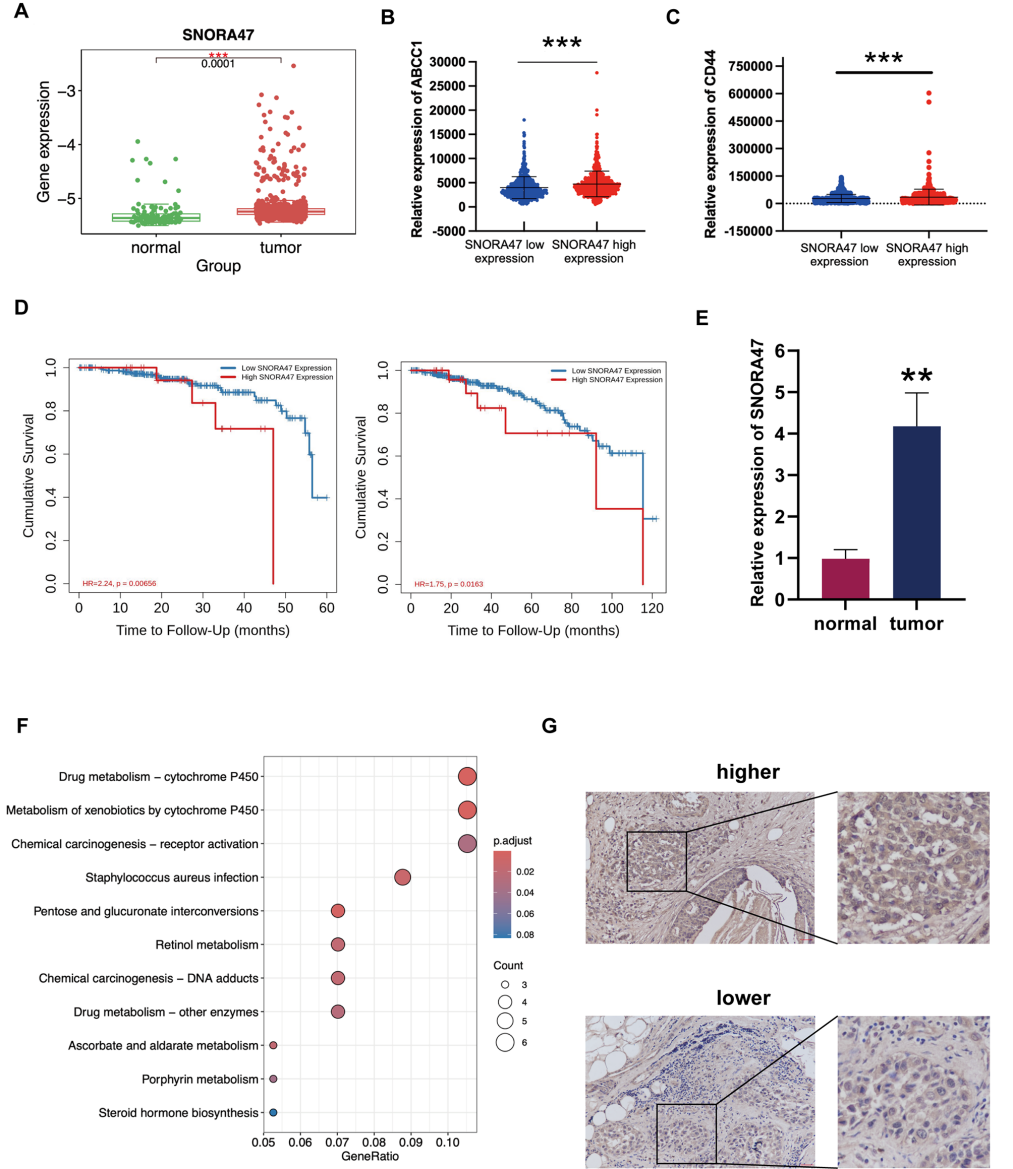
SNORA47 is overexpressed in Luminal A breast cancer. **A**. The expression of SNORA47 in breast cancer was analyzed. **B**. Comparison of relative expression of ABCC1 between SNORA47 high and low expression groups. **C**. Comparison of relative expression of CD44 between SNORA47 high and low expression groups. **D**. The relationship between SNORA47 and survival among Luminal A-subtype breast cancer patients was analyzed via Timer 2.0. **E**. Relative expression of SNORA47 was analyzed. **F**. Visualization of KEGG pathways enriched in the low SNORA47 expression group. The size of each node corresponds to the number of genes involved in the pathway, and the color gradient indicates the level of significance. **G**. ISH analysis of SNORA47 expression levels in Luminal A breast cancer patients, illustrating high and low expression patterns. Scale bar = 50 μm. All the results are displayed as the means ± SDs. *P < 0.05, **P < 0.01, ***P < 0.001, ****P < 0.0001

In our previous work, through snoRNA sequencing (Aksomics, Shanghai, China), we found that SNORA47 was significantly over expressed in MCF-7 MS cells than in MCF-7 (Shan et al. 2024). To explore the expression of SNORA47, we examined the breast cancer dataset from TCGA. Our analytical investigation demonstrated that the transcript levels of SNORA47 were markedly augmented in cancer samples (*n* = 1109) in contrast to normal tissues (*n* = 103) (*P* < 0.0001) (Fig. 1A). Clinical data from the TCGA database were stratified into two groups based on differential SNORA47 expression. KEGG analysis indicated that SNORA47 expression was associated with drug metabolism, metabolism of xenobiotics and chemical carcinogenesis in TCGA breast cancer samples (Fig. 1F). An elevated expression of SNORA47 (median split) was correlated with heightened levels of ABCC1 (*P* < 0.0001) and CD44 (*P* < 0.0001) (Fig. 1B-C). Additionally, results for other drug sensitivity markers (ABCG2, ABCB1, BCL2; *P* < 0.00001) and cancer stem cells (CSCs) markers (ALDH1A1, PROM1; *P* < 0.01) were also presented (Fig. S1E-I). A statistically significant correlation (*P* < 0.01) was observed between an increased expression of SNORA47 (median split) and elevated levels of c-Myc (Fig. S1J).

For the purpose of examining the relationship between the expression levels of SNORA47 and the prognostic outcomes of patients diagnosed with breast cancer, we employed the TIMER 2.0 website (http://timer.cistrome.org). We found higher SNORA47 expression was linked to poorer survival outcomes in patients with Luminal A breast cancer. However, the impact of SNORA47 on the prognosis did not show any statistically significant differences across the other three subtypes (HER2, Basal and Luminal B) (Fig. 1D; Fig. S1K-M). In comparison to the HER2 subtype, the Luminal A subtype exhibited significantly higher expression of SNORA47. Additionally, an increased expression of SNORA47 can be seen in patients who were ER+, PR+, and HER2- demonstrated (*P* < 0.05), via bc-GenExMiner v5.1 (Fig. S1A-D). Therefore, we identified Luminal A breast cancer patients as our focus. We chose thirty pairs of fresh Luminal A tissue samples, along with their corresponding adjacent normal tissues. Via qRT-PCR analysis, our results indicated that SNORA47 was highly expressed in fresh breast cancer tissues (*P* < 0.001) (Fig. 1E).

### SNORA47 is positively associated with TNM stage and tended to have poor NACT response in luminal A breast cancer

To investigate the association between SNORA47 and clinicopathological parameters, this study enrolled 62 Luminal A breast cancer patients. In situ hybridization (ISH) analysis confirmed 47 patients were SNORA47 highly expressed, while 15 were SNORA47 low-expressed (Fig. 1G). High expression of SNORA47 was associated with high TNM stage (*P* = 0.049) (Table 1). To further explore how SNORA47 impact breast cancer drug sensitivity, this study used Luminal A tissues obtained by core needle biopsy before NACT to detect SNORA47 expression. Although without statically significance, high expression of SNORA47 tended to have poor NACT clinical response (*P* = 0.14) and pathological response (*P* = 0.33) (Table S1). These results have provided valuable insights and delineated a strategic direction for our ongoing and future research endeavors.

**Table 1:** Chi-square analysis of SNORA47 and clinical pathology factors

Factors	Number (%)	SNORA47 expression		χ^2^	*P*-value
		Low	High		
Age (years)				0.06	0.800
< 60	36	15	21		
> 61	26	10	16		
Menstruation				0.24	0.628
premenopausal	25	11	14		
postmenopausal	37	14	23		
Family history				1.25	0.264
Yes	12	5	7		
No	40	10	30		
Tumor size (cm)				0.00	0.960
≥ 2	30	12	18		
< 2	32	13	19		
LN Metastases				0.02	0.883
positive	18	7	11		
negative	44	18	26		
Histological grade				0.460	0.498
1–2	29	13	16		
3	33	12	21		
Ki67					
< 30	23	12	11	2.134	0.144
> 31	39	13	26		
TNM staging				6.004	0.049
I	11	8	3		
II	35	11	24		
III	16	6	10		

### SNORA47 affectes stemness and drug sensitivity in luminal A breast cancer cells

**Fig. 2:**
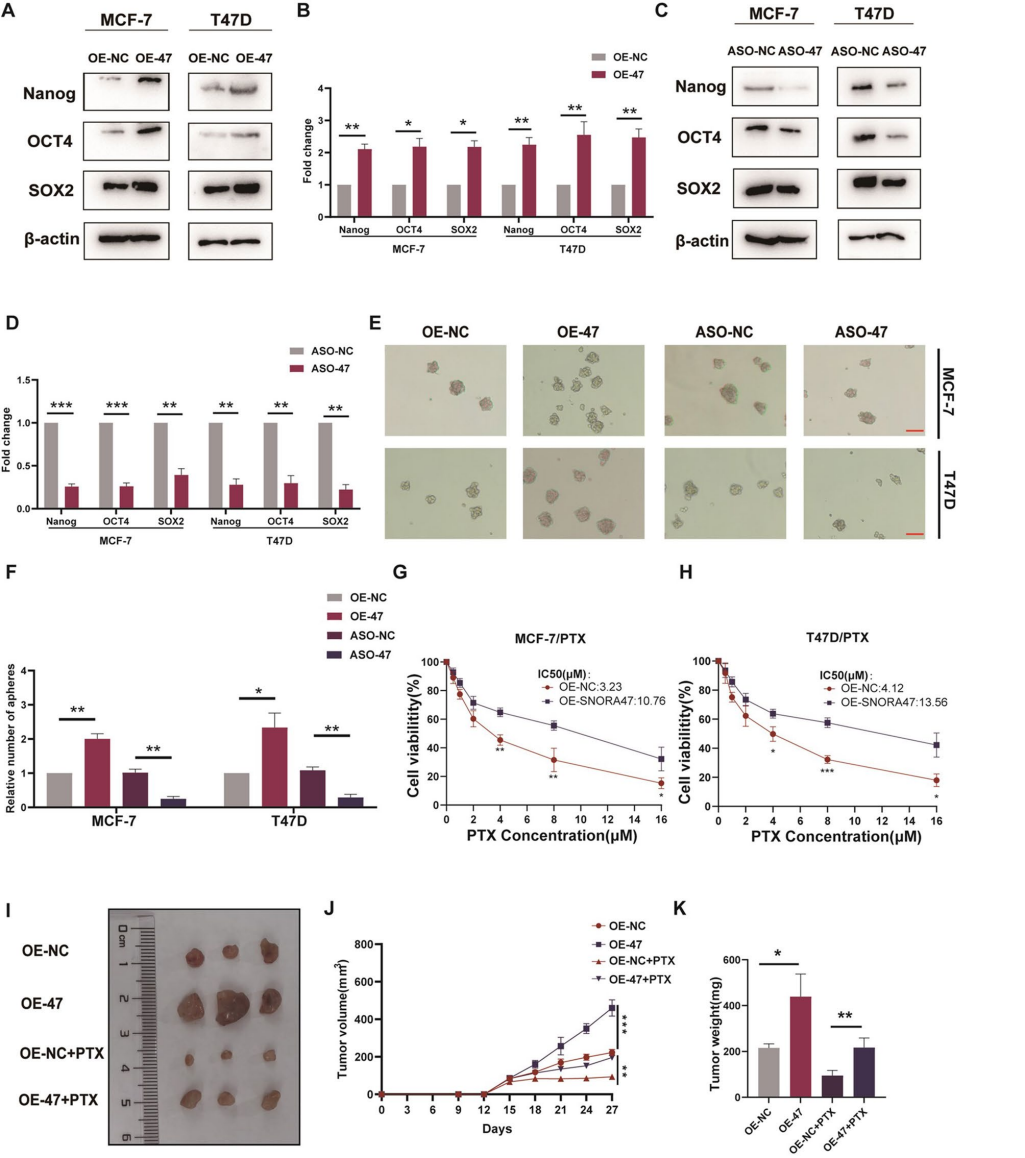
SNORA47 affects stemness phenotype and drug sensitivity in MCF-7 and T47D cells. **A**-**B**. Western blotting analysis of Nanog, OCT4 and SOX2 in MCF-7 and T47D cells after overexpression of SNORA47 (OE-47). **C**-**D**. Western blotting analysis of Nanog, OCT4 and SOX2 in MCF-7 and T47D cells after transfection with anti-SNORA47 ASOs (ASO-47). **E**-**F**. Sphere formation assays in MCF-7 and T47D cells after transfection with SNORA47 or anti-SNORA47 ASOs. Scale bar = 100 μm. G-H. Cell viability and IC_50_ values of different concentrations of paclitaxel in MCF-7 and T47D cells after transfection with SNORA47. **I**. Image of OE-SNORA47 MCF-7 tumor tissues with or without PTX treatment. **J**. Average tumor volumes were measured in xenograft mice. **K**. Image of average tumor weight at the end of indicated treatment. Each experiment was repeated independently at least three times. All the results are displayed as the means ± SDs. *P < 0.05, **P < 0.01, ***P < 0.001, ****P < 0.0001; unpaired two-tailed Student’s t test

To examine the way SNORA47 influences the stemness phenotype and drug responsiveness of breast cancer cells, we transfected SNORA47 and anti-SNORA47 ASOs into MCF-7 and T47D cells and used qRT-PCR to verify changes in SNORA47 expression (Fig. S2A, B). Western blotting analysis revealed that the upregulation of Nanog, OCT4, and SOX2 was observed in breast cancer cells that exhibited overexpression of SNORA47, whereas a downregulation of these markers was evident in cells treated with ASOs targeting SNORA47 (Fig. 2A-D). Interestingly, higher SNORA47 expression in MCF-7 and T47D cells resulted in more tumor spheres than in the vector control, whereas SNORA47-silenced cells formed fewer tumor spheres (Fig. 2E, F). Additionally, the 50% inhibitory concentration (IC_50_) values for SNORA47 up-regulation and down-regulation in response to paclitaxel were measured using CCK-8 assay. The results indicated that the IC_50_ values for paclitaxel were greater in SNORA47-overexpressing MCF-7/PTX (Fig. 2G) and T47D/PTX (Fig. 2H) cells than in control cells. Conversely, the IC_50_ values for paclitaxel were lower in SNORA47-silenced MCF-7/PTX and T47D/PTX (Fig. S2C, D) cells than in control cells. To demonstrate the promotion of MCF-7 cell stemness by SNORA47 in animal models, we injected MCF-7 cells transfected with SNORA47 into nude mice. The overexpression of SNORA47 notably augmented both the size and weight of the tumors (Fig. 2I-K). Ki67 expression is one of the most important markers for assessing tumor cell proliferation in breast cancer (Mitch et al. 2011). In addition to this, we also verified the expression of Ki67 in tumors by immunohistochemistry experiments, which revealed active proliferation in SNORA47 overexpressed tumors in the absence of PTX treatment (Fig. S2E). However, when treated with PTX, SNORA47 did not exhibit significant proliferative capacity, suggesting that SNORA47-mediated tumor enlargement may be related to drug sensitivity. The above results indicate that SNORA47 is closely associated with breast cancer cell stemness and drug sensitivity.

### SNORA47 mediates drug sensitivity and stemness through EBF3 in breast cancer cells

**Fig. 3:**
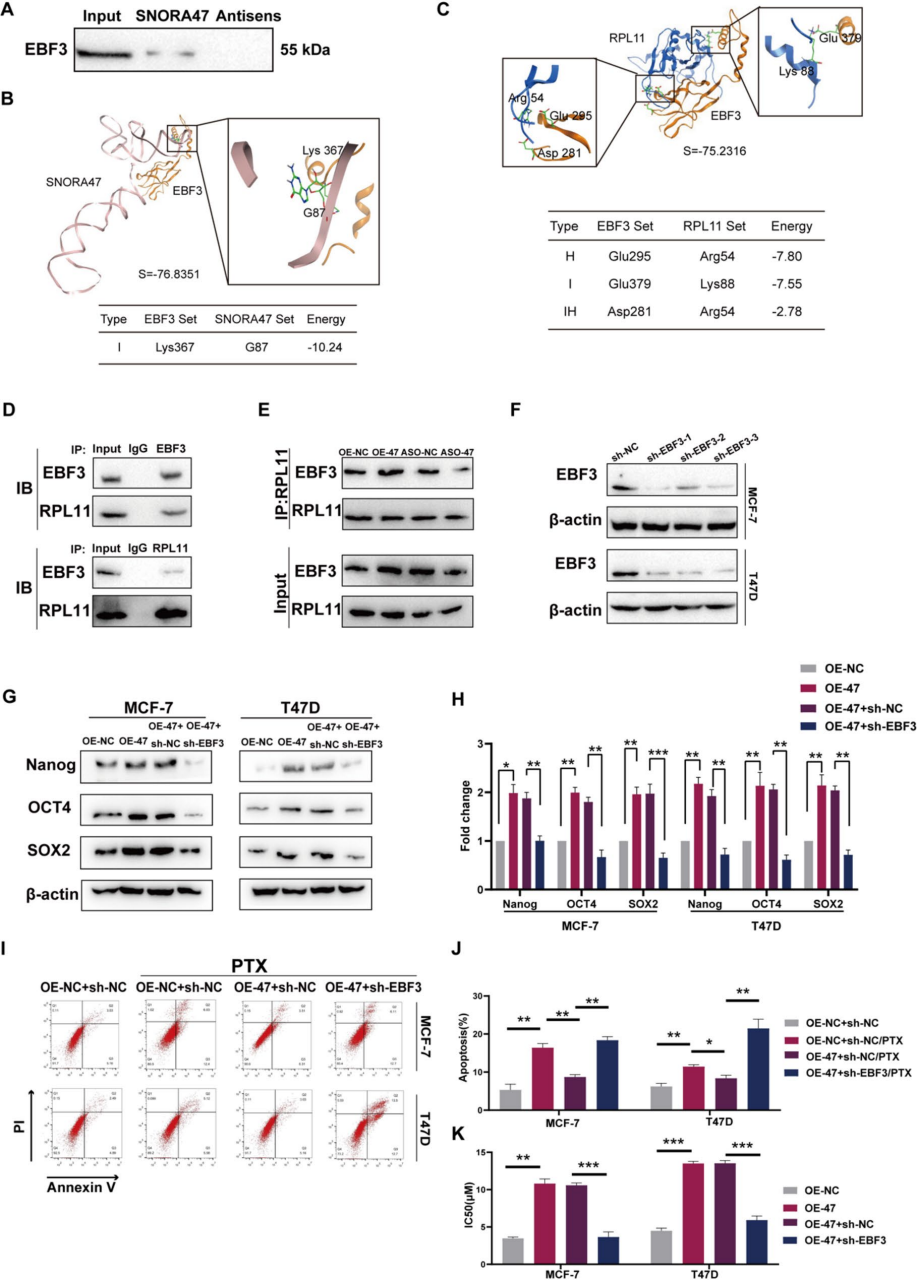
SNORA47 mediates drug sensitivity and breast cancer cell stemness phenotype through EBF3. **A**. Western blotting was performed to validate the presence of specific proteins in the SNORA47-associated complex. **B**. Molecular docking of the SNORA47 and EBF3. **C**. Molecular docking of the RPL11 and EBF3. D-E. Co-IP assay showing that EBF3 interacted with RPL11 (**D**) and changed (**E**) EBF3-RPL11 interactions after transfection with SNORA47 or anti-SNORA47 ASOs in MCF-7 cells. **F**. Western blotting analyses of EBF3 in MCF-7 and T47D cells after EBF3 was knocked down. **G**-**H**. Western blotting analysis of Nanog, OCT4 and SOX2 in MCF-7 and T47D cells. **I**-**J**. Apoptosis of MCF-7 and T47D cells under 2 µM paclitaxel treatment. K. IC_50_ values of paclitaxel in MCF-7 and T47D cells. Each experiment was repeated independently at least three times. All the results are displayed as the means ± SDs. *P < 0.05, **P < 0.01, ***P < 0.001, ****P < 0.0001; unpaired two-tailed Student’s t test

To investigate how SNORA47 affects breast cancer cell stemness and drug sensitivity, we used RNA pull down experiments and searched for proteins that interact with SNORA47 by means of LC-MS (Table S2). Subsequently, we subject the candidates in the mass spectrometry results to molecular docking prediction. We notably used western blotting and the MOE software to analyze the interaction between the SNORA47 and EBF3 (SVM classifier = − 76.8351) (Fig. 3A, B). Several studies have confirmed that EBF3 is associated with various malignant biological behaviours such as proliferation, apoptosis and migration of tumor cells (Kim et al. 2012; Ding et al. 2022a, b, c). Combined with the above results, we speculate that EBF3 could be a crucial molecule in the modulation of the stemness phenotype in breast cancer cells by SNORA47. It has been demonstrated recently that snoRNA family members are closely associated with ribosome proteins (Cheng et al. 2024). Furthermore, molecular docking of EBF3 and RPL11 revealed strong interactions between these two molecules (SVM classifier = − 75.2316) and we observed the enrichment of SNORA47 in EBF3 and RPL11 derived from MCF-7 cells by RIP assay, and further suggested that SNORA47 interacts with EBF3 (Fig. 3C; Fig. S3A). Additionally, our study revealed an interaction between EBF3 and RPL11 in breast cancer cells through Co-IP analysis (Fig. 3D; Fig. S3B). Notably, we observed that SNORA47 potentiated the EBF3-RPL11 interaction, while its functional loss resulted in diminished association (Fig. 3E; Fig. S3C). Therefore, we speculated that SNORA47 could affect RPL11 translocation in the nucleolus and nucleoplasm via EBF3, retaining more RPL11 in the nucleolus. We subsequently transfected an interfering RNA targeting EBF3 into the MCF-7 and T47D cell lines, followed by validation of the mRNA and protein levels (Fig. 3F; Fig. S3D). In contrast to the vector control group, the effective knockdown of EBF3 markedly suppressed the upregulated expression of Nanog, OCT4 and SOX2 in MCF-7 and T47D breast cancer cells that exhibited overexpression of SNORA47 (Fig. 3G-H). In addition, under paclitaxel treatment, SNORA47 cells were more resistant to apoptosis; however, when EBF3 was silenced, the resistance of SNORA47 disappeared (Fig. 3I-K). Therefore, SNORA47 affected the stemness phenotype of breast cancer cells by influencing the interactions between EBF3 and RPL11, which consequently influenced the stemness phenotype and sensitivity of the breast cancer cells.

### SNORA47 promotes c-Myc expression through EBF3-mediated nucleolus-nucleoplasm translocation of RPL11

**Fig. 4:**
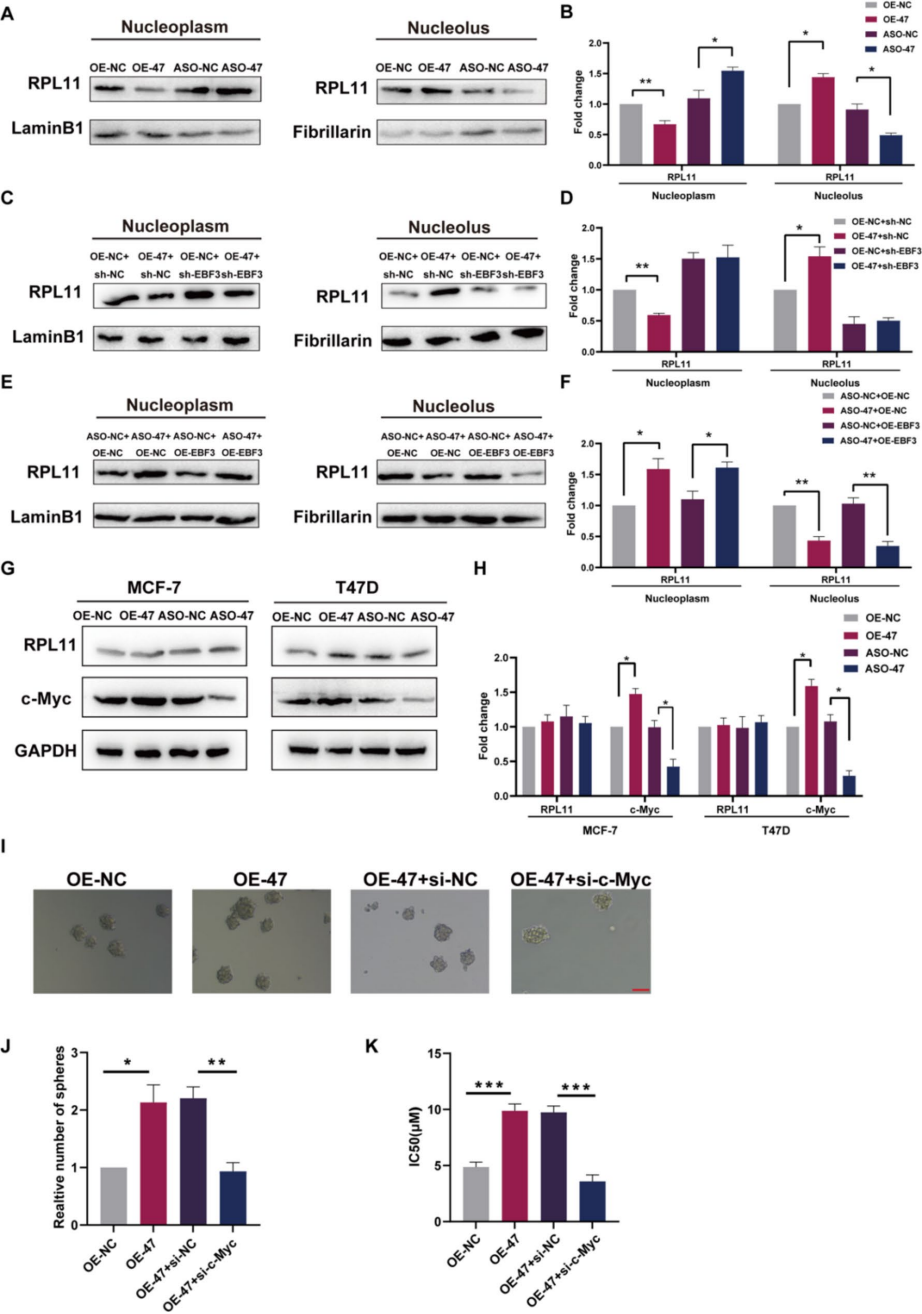
SNORA47 promotes c-Myc expression through RPL11. **A**-**F**. Western blotting analysis of RPL11 in MCF-7 cells after nucleolus-nucleoplasm separation. **G**-**H**. Western blotting analysis of RPL11 and c-Myc in MCF-7 cells.**I**-**J**. Sphere formation assays in MCF-7 cells. Scale bar = 100 μm. **K**. IC_50_ values of paclitaxel in MCF-7 cells. Each experiment was repeated independently at least three times. All the results are displayed as the means ± SDs. *P < 0.05, **P < 0.01, ***P < 0.001, ****P < 0.0001; unpaired two-tailed Student’s t test

To confirm the hypothesis that SNORA47 might influence the translocation of RPL11 within the nucleolus and nucleoplasm through its interaction with EBF3, thereby promoting the retention of a greater amount of RPL11 in the nucleolus, we performed Western blotting analysis after a nucleolus-nucleoplasm separation assay and revealed increased expression of RPL11 within the nucleolus in breast cancer cells overexpressing SNORA47 than in controls, while conversely, SNORA47 expression was elevated within the nucleoplasm in breast cancer cells with silenced SNORA47 (Fig. 4A-B; Fig. S4A-B). To determine whether SNORA47 mediates the different expression profiles of RPL11 in the nucleolus and nucleoplasm via EBF3, we induced knockdown of EBF3 in breast cancer cells that overexpressed SNORA47 and observed that the alterations in the expression levels of RPL11 within the nucleolus and nucleoplasm were abrogated (Fig. 4C-D; Fig. S4C-D). However, the changes in the expression of RPL11 in the nucleolus and nucleoplasm persisted after overexpression of EBF3 in SNORA47-silenced-expressing breast cancer cells (Fig. 4E-F; Fig. S4E-F). RPL11 not only plays a key role in ribosome structure and function, but also regulates c-Myc expression. It has been demonstrated that RPL11 has the capacity to bind to c-Myc mRNA, thereby inducing its degradation. In addition to this, RPL11 has also been shown to enhance the degradation of c-Myc protein, thus leading to a suppression of c-Myc activity (Challagundla et al. 2011; Wang et al. 2022a, b, c). To this end, we suspected that SNORA47 may affect the nucleolus-nucleplasm translocation of RPL11, which in turn promotes c-Myc expression to influence stemness and drug sensitivity of breast cancer cells. A subsequent assessment of c-Myc expression levels within these cells revealed a positive correlation with SNORA47 expression (Fig. 4G-H). Furthermore, silencing EBF3 in SNORA47-overexpressing breast cancer cells resulted in the disappearance of changes in c-Myc expression (Fig. S4 G-H), whereas overexpression of EBF3 did not alter the effect of SNORA47 knockdown on c-Myc expression in breast cancer cells (Fig. S4I-J). These findings suggest that the complex interplay between EBF3 and SNORA47 regulated c-Myc expression and ultimately impacted the malignant phenotype of breast cancer cells. In the following, we investigated whether SNORA47 affects breast cancer cell stemness and drug sensitivity by regulating c-Myc expression. In SNORA47 overexpressing MCF-7 cells, interfering with c-Myc expression results in reduced tumor spheres (Fig. 4I-J; Fig. S4J-K) and IC_50_ values (Fig. 4K). Overall, SNORA47 promoted c-Myc expression and affected breast cancer cell stemness and drug sensitivity through c-Myc.

**Fig. 5:**
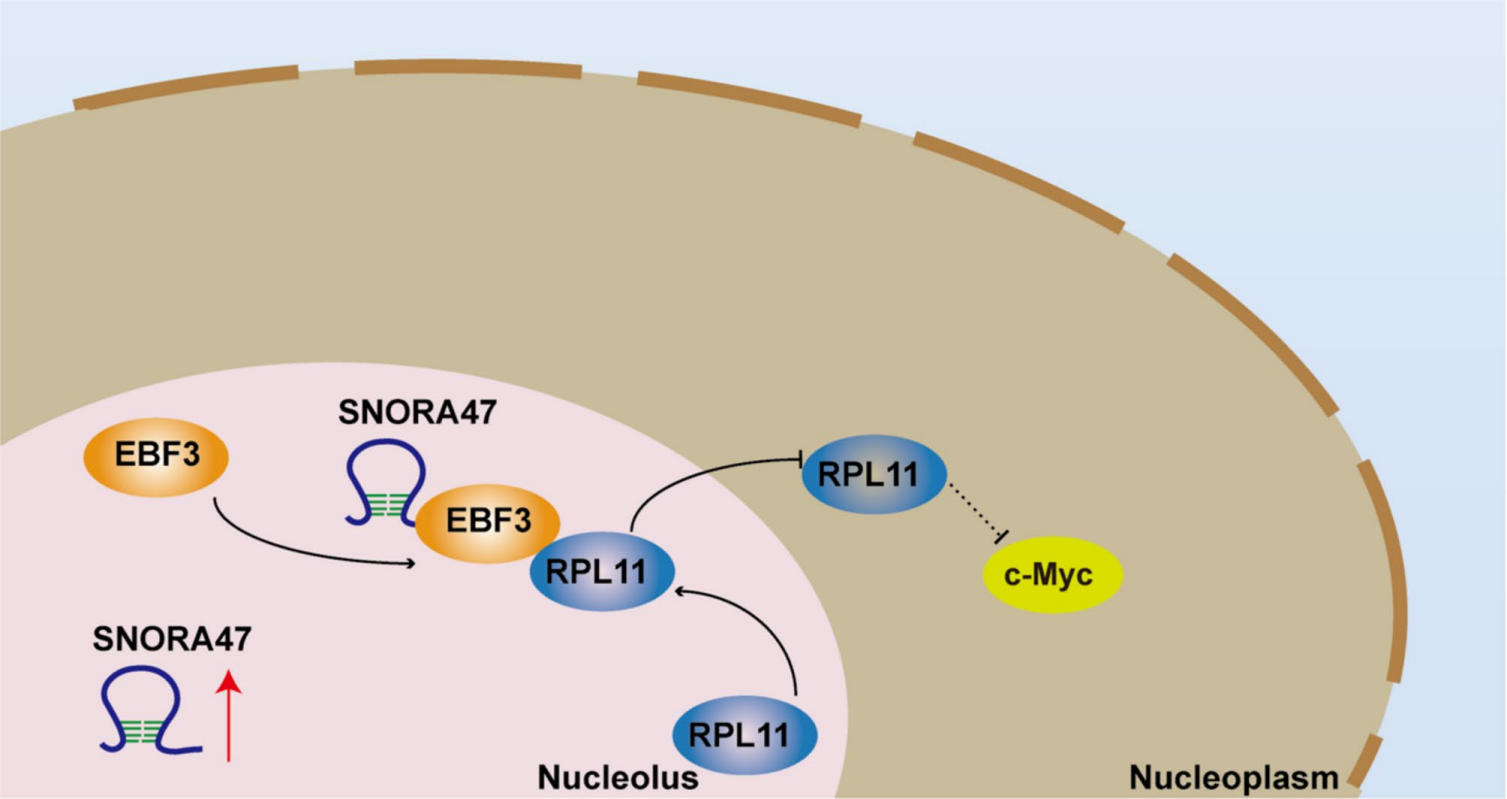
Proposed model for the regulation of c-Myc expression by the SNORA47-EBF3-RPL11 axis. SNORA47 causes RPL11 to localise within the nucleolus by binding to EBF3, which in turn inhibits the down-regulation of c-Myc expression by RPL11, leading to the role of c-Myc in regulating breast cancer cell stemness and drug sensitivity

## Discussion

This research holds potential clinical significance. Patients with Luminal A breast cancer who present with high-risk factors, including lymph node metastasis or elevated genetic risk, may be candidates for adjuvant or neoadjuvant chemotherapy regimens. Although we have more and more endocrine drugs that can reduce recurrence and improve quality of life concurrently, chemotherapy is indispensable for high-risk patients and has a relatively large impact on prognosis. Therefore, once patients are not sensitive to chemotherapy drugs, they can change drugs or endocrine ladder treatment as soon as possible. This study selects 30 Luminal A patients with lymph node metastasis for neoadjuvant chemotherapy, which is conducive to directly observing the relationship between SNORA47 and drug sensitivity.

The potential of snoRNAs as biomarkers for non-small cell lung cancer (NSCLC) has been well established, as exemplified by SNORD33, SNORD66, and SNORD76, which are highly expressed not only in tumors but also in plasma (Jipei et al. 2010). Consequently, these biomarkers play pivotal roles in both diagnosis and prognosis. In our study, bioinformatics analysis has demonstrated that the expression level of SNORA47 is associated with the prognosis of Luminal A breast cancer. Therefore, there is significant promise in validating the stable presence of SNORA47 in blood, its correlation with cancer, and its potential clinical applications, thereby making downstream experiments highly valuable.

The involvement of small nucleolar RNAs (snoRNAs) in cancer has been increasingly acknowledged in recent years. Recent research has demonstrated an association between snoRNAs and localized metastatic events in patients diagnosed with early-stage breast cancer (Escuin et al. 2024). The study identifies a 6-snoRNA signature (SNORD93, SNORD113-6, SNORA7A, SNORA16A, SNORA18A, and SNORA57) that can distinguish between nonmetastatic and metastatic tumors. Patients with low snoRNA expression tended to have improved overall survival (OS) and disease-free survival (DFS). SNORD16, SNORA73B, SCARNA4, and SNORD49B were found to be upregulated in both breast cancer and early-stage breast cancer, and have emerged as potential biomarkers for the diagnosis of these conditions (Li et al. 2024).

However, the precise mechanisms through which they influence oncogenic pathways remain largely uninvestigated. Our discovery of SNORA47’s involvement in the nucleolar-nucleoplasmic shuttling of RPL11 represents a significant contribution to this field of study. The interaction between SNORA47 and EBF3 highlights the importance of transcription factors in snoRNA-mediated regulatory processes. This finding corroborates previous research that identified the multifaceted roles of snoRNAs, which extend beyond their traditional functions in ribosome biogenesis. For example, SNORA18L5 was able to promote ribosomal biosynthesis, increase the maturation of rRNA size subunits, inhibit the entry of RPL5/RPL11 ribosomal proteins into the nucleoplasm to bind to MDM2, and promote the ubiquitylation and degradation of the target P53 to exert the oncogenic effects of snoRNAs in hepatocellular carcinoma cells (Cao et al. 2018). The expression of SNORA71A facilitated the process of EMT in breast cancer cells and correlated with an unfavorable prognosis in individuals suffering from breast cancer (Hu et al. 2022). SNORD33 knockdown increased cell proliferation and decreased apoptosis in TNBC cells. SNORD33 could potentially function as a predictive biomarker for assessing the therapeutic benefit of platinum-based treatments (Wang et al. 2022a, b, c).

EBF3 has been the subject of several studies in tumor cells. EBF3 directly activated p21 and p27 gene transcription and inhibited tumor cell proliferation through cell cycle blockade and apoptosis (Kim et al. 2012). EBF3 promoted nasopharyngeal carcinoma cell migration, invasion and metastasis through Vimentin (Ding et al. 2022a, b, c).Although EBF3 performs different oncogenic or pro-oncogenic functions in tumor cells, the presence of EBF3 in this study connects SNORA47 to RPL11. EBF3 in breast cancer cells should be further explored in the future.

RPL11 not only acted as a protein that played a key role in the structure and function of ribosomes but also regulated cell cycle processes and dictated cell destiny (Tsurugi et al. 1976). RPL11 in the cytoplasm directly interacted with and inhibited the function of MDM2, leading to the stabilization and activation of TP53 (Tong et al. 2020; Lohrum et al. 2003; Zhang et al. 2003; Zheng et al. 2015; Li et al. 2022). In addition, RPL11 is closely related to c-Myc expression. RPL11 repressed c-Myc activity by directly binding to c-Myc mRNA and recruiting RNA-induced silencing complexes (Challagundla et al. 2011; Liao et al. 2014), interacting with c-Myc proteins and competing with transcriptional coactivators to repress c-Myc activity (Wang et al. 2022a, b, c; Jung et al. 2020). As transcriptional targets of c-Myc, RPL5 and RPL11 exhibited the capacity to repress c-Myc activity through the enhancement of c-Myc degradation, thereby operating in a negative feedback mechanism (Dai and Lu 2008; Dai et al. 2007, 2010). Herein, we showed that changes in the translocation of RPL11 in the nucleolus and nucleoplasm may affect changes in c-Myc expression, which in turn affects the stemness phenotype of breast cancer cells. The disruption of this pathway by the SNORA47-EBF3 interaction reveals a previously unidentified means through which breast cancer cells can manipulate c-Myc to their advantage.

Importantly, the affected CSC characteristics and drug sensitivity exhibited by breast cancer cells with elevated SNORA47 expression indicate that this snoRNA may serve as a prospective therapeutic objective. CSCs are characterized by their capacity for self-renewal and differentiation, which contributes to tumor heterogeneity and resistance to conventional therapies. Inhibition of the SNORA47-EBF3-RPL11 axis may constitute an effective strategy for disrupting these processes, reducing CSC populations and increasing tumor sensitivity to treatment. Future research endeavors should concentrate on validating these observations in clinical specimens and investigating the potential therapeutic implications of targeting SNORA47 and its cognate pathways.

In summary, these findings suggest that targeting SNORA47-EBF3-RPL11/c-Myc pathway can influence CSC properties and drug sensitivity. These discoveries augment our comprehension of the molecular mechanisms driving breast cancer and suggest that SNORA47 could serve as a potential drug susceptibility marker.

### Experimental methodology

#### Patients and clinical specimens

30 breast cancer patients hospitalized at the First Hospital of China Medical University from Jan. to Mar. 2023, were selected for this study. The criteria for inclusion were stipulated as follows: (I) Patients with the Luminal A subtype who were undergoing NACT; (II) Tissues obtained through core needle biopsy prior to NACT were available; (III) Detailed documentation encompassing the evaluation results obtained at intervals of every two treatment cycles was maintained. Immediately post-biopsy freshly obtained Luminal A tissues, and their corresponding adjacent normal tissues (*n* = 30 pairs) were rapidly frozen in liquid nitrogen and subsequently stored for potential future analytical purposes. The clinical response to NACT was assessed in accordance with the Response Evaluation Criteria in Solid Tumors (RECIST 1.0).

Samples embedded in paraffin (*n* = 62 patients) were amassed from individuals hospitalized between January and December in 2017, all of whom had received a diagnosis of Luminal breast carcinoma. Sections of paraffin were procured from the primary breast malignancies. Subsequent follow-up data were collected either directly from the patients or through their closest relatives. The Ethical Review Board of China Medical University granted approval for this research endeavor (Approval number: AF-SOP-07-1.1-01).

### Cell culture and transfection procedures

We selected MCF-7 and T47D (Human breast cancer cell lines) as our target, which acquired from the Cell Bank, Chinese Academy of Sciences. The MCF-7 and T47D breast cancer cell lines serve as exemplary models for the Luminal A subtype, distinguished by their specific gene expression patterns and associated clinical attributes (Deborah and Valerie 2011). Each of these cell lines underwent a comprehensive screening process for mycoplasma contamination, DNA fingerprinting, isozyme analysis, and assessment of cell viability. This screening was executed by an impartial biological services enterprise (GeneCreate Biology Co., Ltd., Wuhan, China). The MCF-7 cell line was cultivated in MEM medium that was supplemented with 10% fetal bovine serum (FBS) and 10 µg/mL of insulin. Conversely, the T47D cell line was sustained in RPMI-1640 medium within a controlled environment incubator maintained at 37 °C and supplemented with 5% CO2.

SNORA47 and EBF3 vector were purchased from Sango Biotech Co.,Ltd. (Shanghai, China). ShRNAs against EBF3 was acquired from Genechem Co.,Ltd. (Shanghai, China). Transfection of shRNA oligonucleotide and plasmids were performed with Lipofectamine 3000 (Invitrogen). The sequence of EBF3 shRNAs was as follows: shEBF3-1: 5’-CACCGCCCACCGTCATCATAATTCTCGAGAATTATGATGACGGTGGCACCTTTTTTG-3’, shEBF3-2: 5’-CACCGCCGGCTACAGTCGCAATACCTCGAGGTATTGCGACTGTAGCCGACTTTTTTTG-3’. shEBF3-3: 5’-CACCGCGCCTCATAGATTCAATGCTCGAGCATTGAATCTATGAGGCGAACTTTTTTG-3’.ASO-SNORA47 was procured from RiboBio Co.,Ltd. (Guangzhou, China). Small interfering RNAs (siRNAs) targeting c-Myc were synthesized by our commission from Genechem Co.,Ltd. (Shanghai, China). The siRNA sequence is as follows: 5’-GGAAGAAAUCGAUGUUGUUTT-3’. The transfection procedure was conducted on the cells once they had reached a confluence of 40%, employing the X-tremeGENE siRNA Transfection Reagent (catalog number: 04476093001; Roche Diagnostics GmbH, Mannheim, Germany). The final concentration of the antisense oligonucleotide (ASO) was established at 60 nM. The specific sequence targeted by ASO-h-SNORA47_001 was determined to be AATTTGGAGGTTCCACAACT.

### Sphere formation assay

A sphere formation assay was conducted in accordance with previously established and standardized protocols (Shan et al. 2024). In summary, MCF-7 and T47D cell suspensions (1 × 10³ cells per well) were planted into ultralow adhesion plates (Corning, Kraemer, CA). The cellular cultures were maintained in 2 mL of serum-depleted DMEM-F12 medium, which was fortified with 2% B27 (Absin, China), 10 µg/L bFGF (Sino Biological, China), and 20 µg/L EGF (Sino Biological, China).

### Induction and culture of MCF-7 MS cells

MCF-7 MS cells (MCF-7 mammosphere cells), a type of non-adherent breast cancer stem cell model, grow in suspension without serum or adhesion, forming globular clusters of 200–500 cells and is considered a kind of in vitro model of breast cancer stem cells. MCF-7 MS cells were induced and cultured in a mature process, as previously described (Shan et al. 2024). MCF-7 cells were washed with PBS buffer, digested, and centrifuged. The supernatant was discarded, and the cells were again washed with PBS buffer and centrifuged at 800 rpm for 5 min. They were resuspended in DMEM/F12 medium (containing 2% B27, b-FGF 10 µg/L, EGF 20 µg/L), then transferred to non-adherent bottles and incubated. After 3–4 passages, spherical clusters formed. Expression of SOX2, Nanog, and OCT4 was detected by qRT-PCR and Western blot, while the CD44 (+)CD24 (-) cell population was analyzed by flow cytometry.

### Cell counting Kit-8 (CCK-8) assay

Cell viability was ascertained utilizing a CCK-8 assay kit (Dojindo). The cells (5,000 cells/well) were plated in 96-well ultralow adhesion plates. To determine the IC_50_ values, cells were treated with different concentrations of epirubicin for 48 h. Next, the cells in each well were incubated with 10 µl of WST-8 at 37 °C for 4 h. The optical density (OD) was then measured at 450 nm via an Anthos 2010 microplate reader (Anthos LabtecInstruments GmbH, Austria).

### Cell fractionation

Succinctly, a cellular suspension of 1 × 10⁷ cells was prepared in 500 µL of a hypotonic buffer (10 mM HEPES [pH 7.9], 10 mM KCl, 1.5 mM MgCl₂, and 0.5 mM DTT). The suspension was vigorously vortexed and shaken at 4 °C for 10–30 min. Following centrifugation at a speed of 2,000 × g for a duration of 5 min, the supernatant was collected to comprise the cytoplasmic fraction. Subsequently, the residual pellet was resuspended in S1 buffer, which comprised 10 mM MgCl₂ and 0.25 M sucrose. This suspension was then layered onto 300 µL of S2 buffer containing 0.5 mM MgCl₂ and 0.35 M sucrose, followed by centrifugation at 1,500 × g for a duration of 5 min. To isolate the nuclear fraction, the pellet was resuspended in S2 buffer. This nuclear fraction was sonicated in short bursts (2-second pulses with 3-second intervals) for 6–7 min, then layered onto S3 buffer (0.05 mM MgCl₂, 0.88 M sucrose). After undergoing centrifugation at 3,000 × g for 10 min, the supernatant was designated as the nucleoplasmic fraction. Following its resuspension in RIPA buffer, the pellet served as the nucleolar fraction. Subsequently, all fractions underwent SDS-PAGE for subsequent immunoblotting (IB) analysis.

### RNA pull-down assay

Following the lysis of 2 × 10^7^ breast cancer cells in 1 mL of RNA immunoprecipitation (RIP) buffer, cellular lysates were isolated through centrifugation at 13,000 × g for 10 min at a temperature of 4 °C. Biotin-labeled RNA probes specific to SNORA47 were incubated with the obtained cell lysates for 6 h at 37 °C. Subsequently, 50 µL of washed streptavidin-coated magnetic beads sourced from Invitrogen were introduced to each binding reaction and incubated for 1 h at room temperature. The beads underwent three washing steps using RIP washing buffer, followed by their collection. Ultimately, the collected beads were analyzed via Western blotting.

### RNA Immunoprecipitation (RIP)

The Magna RIP RBP Immunoprecipitation Kit (EMD Millipore, Germany) was used to perform the RIP procedure according to the manufacturer’s instructions. In brief, 2 × 10^7^ cells were collected and lysed with RIPA lysis buffer (Beyotime, China). Following the process of centrifugation, the upper layer of the mixture was subjected to immunoprecipitation, a process in which specific antibodies and protein A/G magnetic beads were employed. The conjugate, which had been bound to the magnetic beads, was then immobilised using a magnet. Thereafter, unbound components were washed off. Finally, RNA bound to the A/G beads was obtained. The real-time qPCR procedure was performed to detect SNORA47 in the precipitates.

### Coimmunoprecipitation (Co-IP)

A quantity of 2 × 10^7^ cells was harvested and lysed in 1 mL buffer (1% Triton X-100, 1 mM EDTA, 50 mM Tris-HCl, 150 mM NaCl, 10% glycerol and protease inhibitor mixture, pH 7.4). Cellular lysates were acquired through centrifugation conducted at 13,000 rpm for a duration of 20 min at 4 °C. Prior to incubation, the lysates were pre-cleared with protein G-conjugated agarose (GE Healthcare Life Sciences) for a period of 4 h. Thereafter, the lysates underwent incubation with the antibodies at 4 °C overnight. Thereafter, the cellular lysates were subjected to immunoprecipitation utilizing protein G-conjugated agarose for an additional 4 h at 4 °C, followed by washing with IP-wash buffer (1% Triton X-100, 1 mM EDTA, 50 mM Tris-Cl, 300 mM NaCl, pH 7.4).

### Molecular operating environment (MOE)

The MOE (Chemical Computing Group, Montreal, QC, Canada) RNA‒Protein Dock was used for SNORA47 and EBF3 interaction. We used 3dRNA (http://biophy.hust.edu.cn/new/3dRNA) to predict the crystal structure of SNORA47. The MOE Protein‒Protein Dock was used for the interaction between the EBF3 and RPL11. The crystal structures of the EBF3 (PDB ID:3N50) and RPL11 (PDB ID: 8A3D) were obtained from the RCSB PDB (https://www.wwpdb.org/) (accessed on 30 July 2024). Finally, we performed100 poses of 2 proteins and chose the lowest energy pose to represent the strongest binding.

### TCGA analysis

The edgeR package, obtained from the TCGA-BRCA database (https://cancergenome.nih.gov/) and used within the R environment, was applied for the normalization of gene expression data. To ascertain the biological functions and pathways impacted by the differentially expressed genes (DEGs), a KEGG pathway enrichment analysis was executed utilizing the Database for Annotation, Visualization, and Integrated Discovery (DAVID) tool (https://david.ncifcrf.gov/) within the R environment.

### RNA extraction and quantitative real-time PCR (qRT-PCR)

RNA extraction and qRT-PCR procedures were executed in accordance with previously described methods (Shan et al. 2024). Furthermore, the extraction of snoRNA from plasma and the quantitation of plasma SNORA47 expression via qRT-PCR were carried out following a previously published protocol. The primer sequences are listed below: SNORA47: Forward 5’-GGAGGACTGAGAAGGTGAGGC-3’ Reverse 5’-GGCAAGGGGACATCCTCTG-3’ EBF3: Forward 5’-GAAAGAGCCAAACAACGAG-3’ Reverse 5’-ATGATTACAGGGTCTGAGGG-3’ c-Myc: Forward 5’-GTCAAGAGGCGAACACACAAC-3’ Reverse 5’- TTGGACGGACAGGATGTATGC-3’β-actin Forward 5’-GTCCACCGCAAATGCTTCTA-3’ Reverse 5’-TGCTGTCACCTTCACCGTTC-3’.

### Flow cytometry

Cells treated by trypsin were resuspended in binding buffer, and then stained with 5 µL Annexin V-FITC and PI at 37 °C for 30 min in the dark. Flow cytometric analysis was performed on a MACSQuantTM flow cytometer. a MACSQuant™ Flow Cytometer (Miltenyi Biotec).

### Western blotting

Western blotting was conducted in accordance with previously reported methods (Shan et al. 2024). For isolating nuclear and cytoplasmic fractions, the NE-PER™ Nuclear and Cytoplasmic Extraction Reagents Kit (manufactured by Thermo Scientific, USA) was employed. The membrane underwent staining with a primary antibody targeted against beta Actin (1:1000,Cell signaling technology, 4947), GAPDH (1:1000,abcam, ab8245), c-Myc (1:1000, abcam, ab185656), EBF3 (1:1000, abcam, ab207755; ab154019), RPL11 (1:1000, Cell signaling technology, 18163; 1:1000, abcam, ab318976), Nanog (1:1000, Cell signaling technology, 4903), OCT4 (1:1000, Cell signaling technology, 2750), SOX2 (1:1000, Cell signaling technology, 3579), Fibrillarin (1:1000, abcam, ab4566) and Lamin B1 (1:1000, abcam, ab16048). Secondary antibodies are HRP-Goat anti Rabbit (1:1000, Abbkine, A25222; A25022), HRP-Donkey anti mouse (1:10000, Cell signaling technology, 7076) and HRP-Donkey rabbit (1:1000, Cell signaling technology, 7074).

### In situ hybridization (ISH) and immunohistochemistry (IHC)

In situ hybridization (ISH) and immunohistochemistry (IHC) were carried out as described in previous studies (Shan et al. 2024). Following the snoRNA ISH Kit (Boster) protocol, the sequence of the target gene SNORA47 was identified as 5’-TGGAGGACTGAGAAGGTGAGGCAGTTTTGCCCCGTGCTGC-3’. The expression level of SNORA47 was assessed using a semi-quantitative double-score method, with at least ten fields on each slide analyzed and 100 cells examined under 400× magnification. Staining intensity was rated on a scale of 0 (negative) to 3 (intense), and the percentage of positive cells was also categorized into five levels, from 0 (< 5%) to 4 (> 76%). The overall ISH score was derived by multiplying the two values. Subsequently, patients were categorized into two distinct groups based on their expression levels of SNORA47: SNORA47-high (score > 3) and SNORA47-low (score ≤ 3). An Ultrasensitive™ S-P Kit (Maixin-Bio, China) was employed for this process.

For the IHC, briefly, Ultra-sensitive™ S-P Kit (Maixin-Bio, China) was used and paraffin-embedded tumor tissue sections from transplanted nude mice were incubated with Ki67 primary antibody (1:100, abcam, ab15580). Results were evaluated by two separate pathologists who were blinded to the experiments.

### Xenograft model

A total of 1 × 10^6^ MCF-7 cells transfected with SNORA47 were suspended in 100 µl of PBS and then injected subcutaneously into 4-week-old male BALB/c nude mice (Beijing Huafu Kang Biotechnology Co., Ltd.). When the tumour had grown roughly to approximately 100 mm^3^, PTX (10 mg/kg) was pre-dissolved in PBS and injected intraperitoneally every 3 days, and the control group was injected with an equal amount of PBS. The diameter and weight of the tumors were assessed at intervals of three days following tumor formation. All mice were maintained in a pathogen-free environment within the animal facility. All animals were housed in facilities with suitable temperature and humidity and had a 12-hour light-­dark cycle. Food and water aread libitum.

### Statistical analysis

Statistical analyses were performed using GraphPad Prism version 8.0 (La Jolla, CA, USA). Data are presented as means ± standard deviations derived from at least three independent experiments. For group comparisons, Student’s t-test was utilized, while Pearson’s chi-square test was applied to analyze the associations between SNORA47 expression and clinicopathological characteristics. A p-value that is less than 0.05 is deemed to be statistically significant.

## Electronic supplementary material

Below is the link to the electronic supplementary material.

Supplementary Material 1: Fig. S1: SNORA47 is associated with drug sensitivity and stemness genes (A) SNORA47 was highly expressed in Luminal A subtype, compared with the HER2 subtype, via bc-GenExMiner. (B) Comparison of relative expression of SNORA47 between ER + and ER- breast cancer groups. (C) Comparison of relative expression of SNORA47 between PR + and PR- breast cancer groups. (D) Comparison of relative expression of SNORA47 between HER2 + and HER2- breast cancer groups. (E) Comparison of relative expression of ABCG2 between SNORA47 high and low expression groups. (F) Comparison of relative expression of ABCB1 between SNORA47 high and low expression groups. (G) Comparison of relative expression of BCL2 between SNORA47 high and low expression groups. (H) Comparison of relative expression of ALDH1A1 between SNORA47 high and low expression groups. (I) Comparison of relative expression of PROM1 between SNORA47 high and low expression groups. (J) Comparison of relative expression of MYC between SNORA47 high and low expression groups. K. The relationship between SNORA47 and survival among Her2-subtype breast cancer patients was analyzed via Timer 2.0. L. The relationship between SNORA47 and survival among Basal-subtype breast cancer patients was analyzed via Timer 2.0. M. The relationship between SNORA47 and survival among Luminal B-subtype breast cancer patients was analyzed via Timer 2.0

Supplementary Material 2: Fig.S2.Silencing SNORA47 expression enhances drug sensitivity in breast cancer cells. A. qRT-PCR analysis of SNORA47 in MCF-7 and T47D cells after overexpression of SNORA47 (OE-47). B. qRT-PCR analysis of SNORA47 in MCF-7 and T47D cells after transfection with anti-SNORA47 ASOs (ASO-47). C-D. Cell viability and IC50 values of different concentrations of paclitaxel in MCF-7 and T47D cells after transfection with anti-SNORA47 ASOs. E. Ki67 protein levels were detected in tumor tissues with or without PTX treatment by IHC. Scale bar =50 m. All the results are displayed as the means SDs. *P < 0.05, **P <0.01, ***P < 0.001, ****P <0.0001; unpaired two-tailed Student’s t test.

Supplementary Material 3: Fig.S3. SNORA47 promotes interactions between EBF3 and RPL11.A. RNA immunoprecipitation (RIP) was performed using IgG, EBF3 and RPL11 antibodies with lysates from MCF-7 cells. Enrichment of SNORA47 was measured using qRT-PCR. B-C. Co-IP assay showing that EBF3 interacted with RPL11 (B) and changed (C) EBF3-RPL11 interactions after transfection with SNORA47 or anti-SNORA47 ASOs in T47D cells. D. qRT-PCR analyses of EBF3 in MCF-7 and T47D cells after EBF3 was knocked down. Each experiment was repeated independently at least three times. All the results are displayed as the means SDs. *P < 0.05, **P < 0.01, ***P < 0.001, ****P <0.0001; unpaired two-tailed Student’s t test.

Supplementary Material 4: Fig.S4. SNORA47 affects c-Myc expression via EBF3.A-F. Western blotting analysis of RPL11 in T47D cells after nucleolus-nucleoplasm separation. G-J. Western blotting analysis of RPL11, EBF3 and c-Myc in MCF-7 and T47D cells. K. qRT-PCR analyses of c-Myc in MCF-7 cells after c-Myc was knocked down. L. Western blotting analyses of c-Myc in MCF-7 cells after c-Myc was knocked down. Each experiment was repeated independently at least three times. All the results are displayed as the means SDs. *P < 0.05, **P <0.01, ***P < 0.001, ****P <0.0001; unpaired two-tailed Student’s t test.

Supplementary Material 5: Table S1. The relationship of SNORA47 and NACT response

Supplementary Material 6: Table S2. Candidate proteins identified by RNA pull-down/LC-MS assays.

## Data Availability

No datasets were generated or analysed during the current study.

## Declarations

### Access to data and materials

The datasets utilized and/or subjected to analysis during this study can be obtained from the corresponding author upon submission of a reasonable request.

### Authorization for publication

All authors have consented to the publication of this article.

## Abbreviations

ASO: Antisense Oligonucleotides
BCSCs: Breast Cancer Stem Cells
bFGF: Basic Fibroblast Growth Factor
CCK-8: Cell Counting Kit-8
Co-IP: Co-Immunoprecipitation
CSC: Cancer Stem Cell
DFS: Disease-Free Survival
EBF3: Early B-cell Factor 3
EGF: Epidermal Growth Factor
EMT: Epithelial–Mesenchymal Transition
FBL: Fibrillarin
HER2: ErbB2 Receptor
ISH: In Situ Hybridization
KEGG: Kyoto Encyclopedia of Genes and Genomes
MOE: Molecular Operating Environment
MS: Mammosphere
OE: Over Expression
qRT-PCR: quantitative Real-Time PCR
RIP: RNA Immunoprecipitation
SD: Standard Deviation
SnoRNAs: Small Nucleolar RNAs
