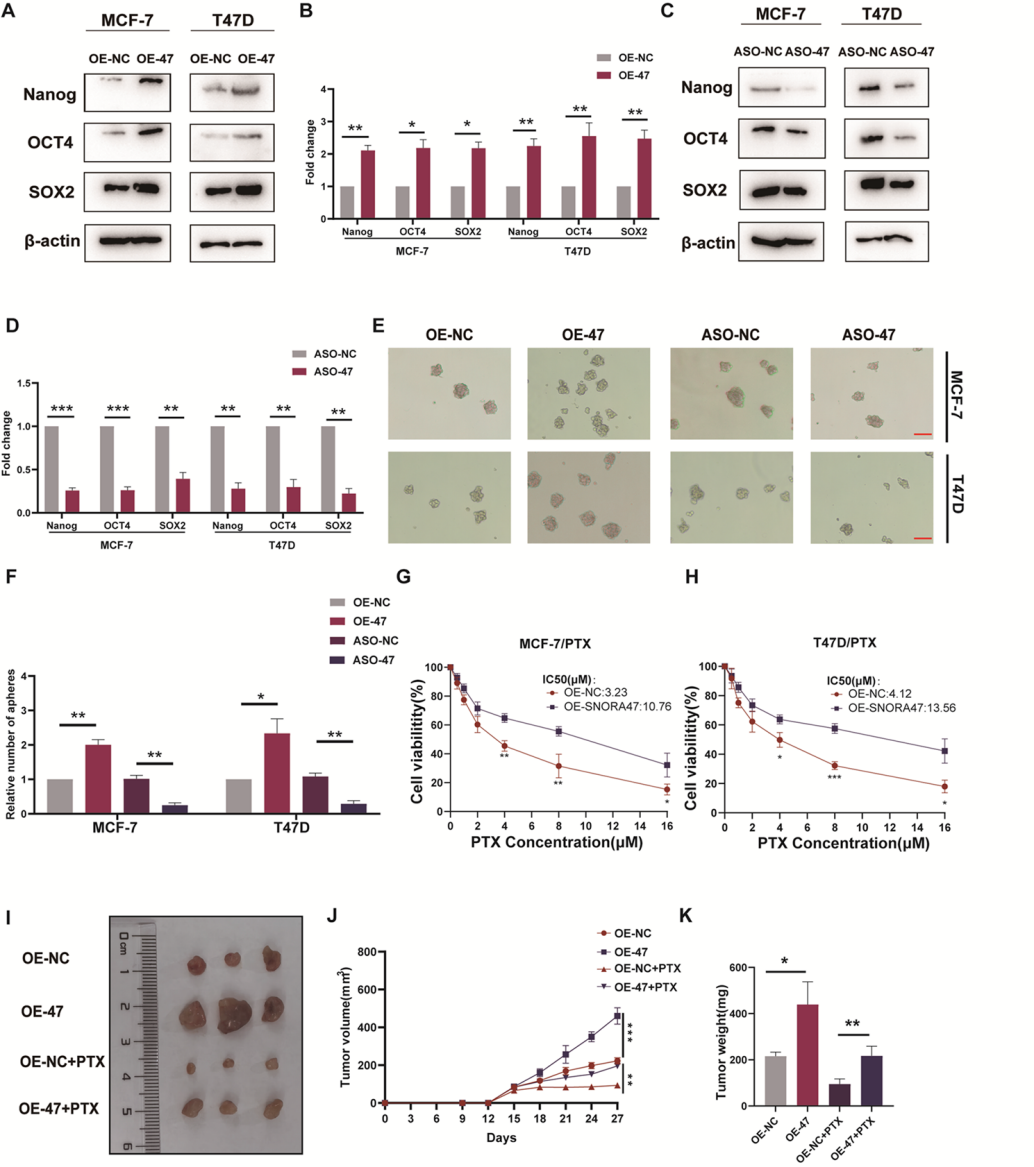# Correction: SNORA47 affects stemness and chemotherapy sensitivity via EBF3/RPL11/c-Myc axis in luminal A breast cancer

**DOI:** 10.1186/s10020-025-01276-5

**Published:** 2025-06-04

**Authors:** Qilin Han, Ying Zhou, Zixian Dong, Weitao Wang, Menghan Wang, Mengyang Pang, Xinyue Song, Bo Chen, Ang Zheng

**Affiliations:** 1 Department of Breast Surgery, the First Hospital of China Medical University, 155 Nanjing North Street, Heping District, Shenyang, Liaoning 110001 China; 2 College of Life Science, China Medical University, Shenyang, China; 3 Department of Pharmacology, School of Pharmacy, China Medical University, Shenyang, China

**Correction: Mole Med 31, 150 (2025)**

**https://doi.org/10.1186/s10020-025-01216-3**

In this article (Han et al. 2025), Fig. 2 appeared incorrectly and have now been corrected in the original publication. For completeness and transparency, both correct and incorrect versions are displayed below.

The original article has been corrected.

Incorrect Figure 2


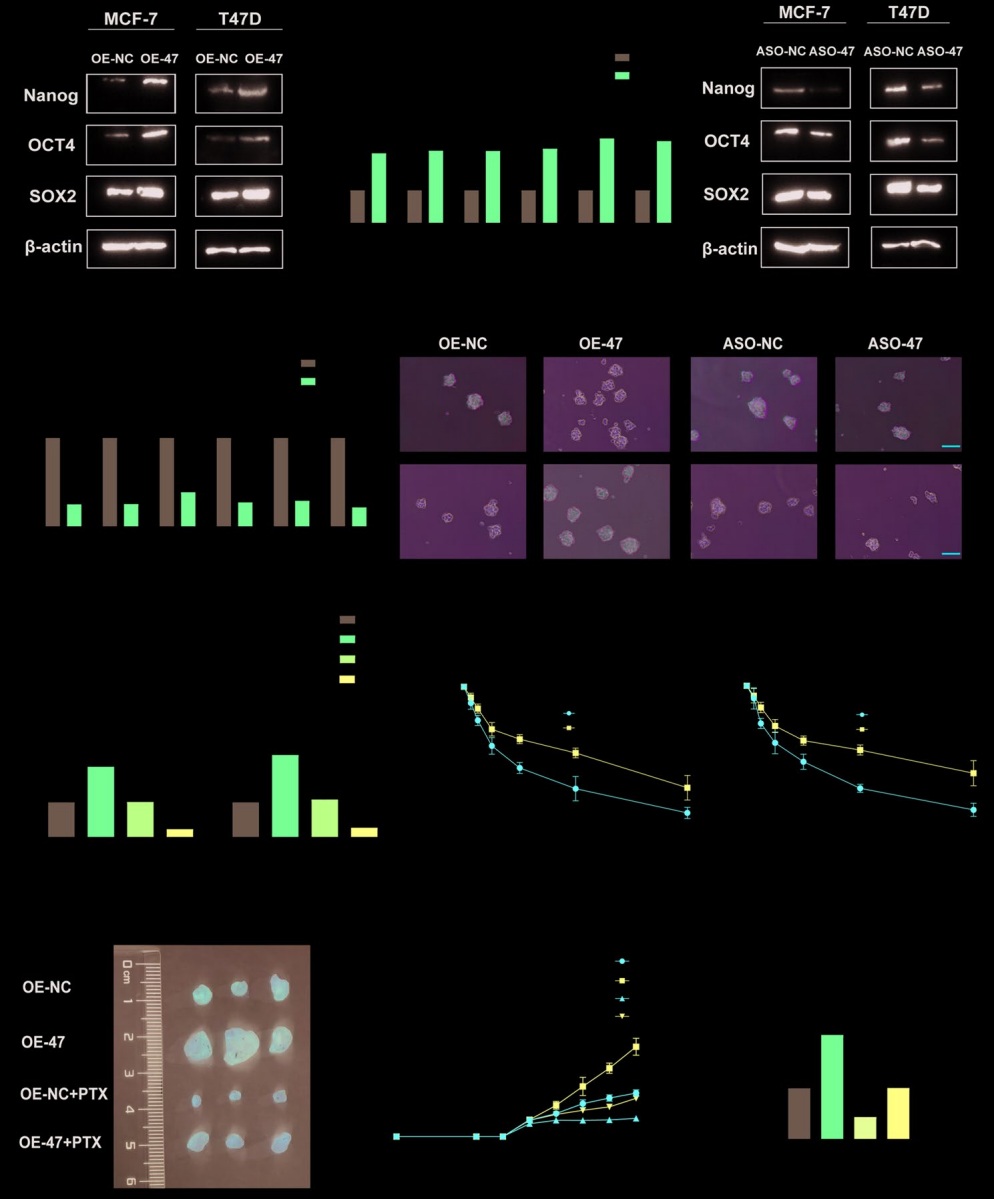


Correct Figure 2