# Supplementary figures and images for: SNORA47 affects stemness and chemotherapy sensitivity via EBF3/RPL11/c-Myc axis in luminal A breast cancer
Source: Mol Med. 2025 Apr 22;31:150. doi: 10.1186/s10020-025-01216-3 (PMC12016144; doi:10.1186/s10020-025-01216-3)

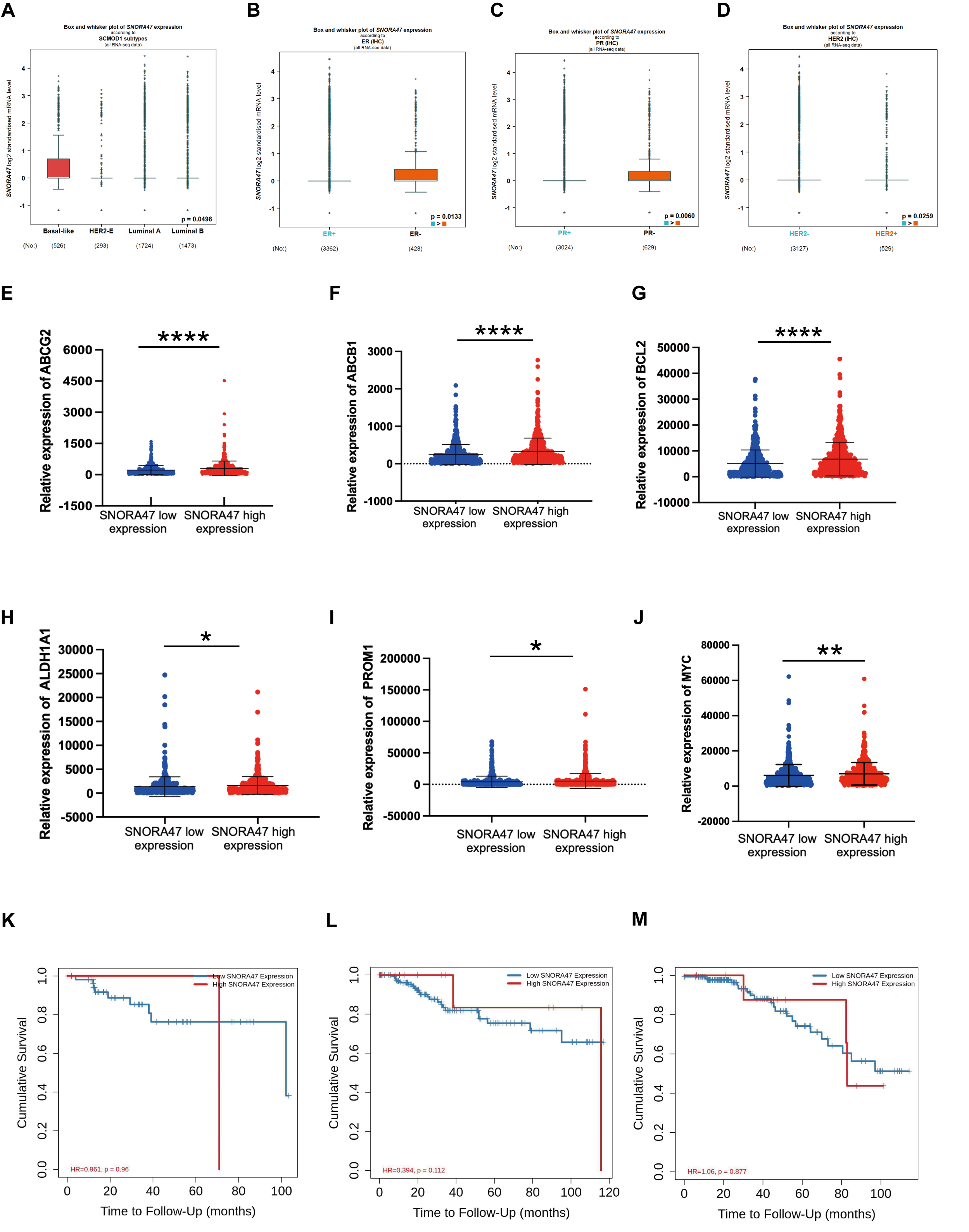

Supplement: Supplementary file 1 — Supplementary Material 1: Fig. S1: SNORA47 is associated with drug sensitivity and stemness genes (A) SNORA47 was highly expressed in Luminal A subtype, compared with the HER2 subtype, via bc-GenExMiner. (B) Comparison of relative expression of SNORA47 between ER + and ER- breast cancer groups. (C) Comparison of relative expression of SNORA47 between PR + and PR- breast cancer groups. (D) Comparison of relative expression of SNORA47 between HER2 + and HER2- breast cancer groups. (E) Comparison of relative expression of ABCG2 between SNORA47 high and low expression groups. (F) Comparison of relative expression of ABCB1 between SNORA47 high and low expression groups. (G) Comparison of relative expression of BCL2 between SNORA47 high and low expression groups. (H) Comparison of relative expression of ALDH1A1 between SNORA47 high and low expression groups. (I) Comparison of relative expression of PROM1 between SNORA47 high and low expression groups. (J) Comparison of relative expression of MYC between SNORA47 high and low expression groups. K. The relationship between SNORA47 and survival among Her2-subtype breast cancer patients was analyzed via Timer 2.0. L. The relationship between SNORA47 and survival among Basal-subtype breast cancer patients was analyzed via Timer 2.0. M. The relationship between SNORA47 and survival among Luminal B-subtype breast cancer patients was analyzed via Timer 2.0 [file 10020_2025_1216_MOESM1_ESM.tif]

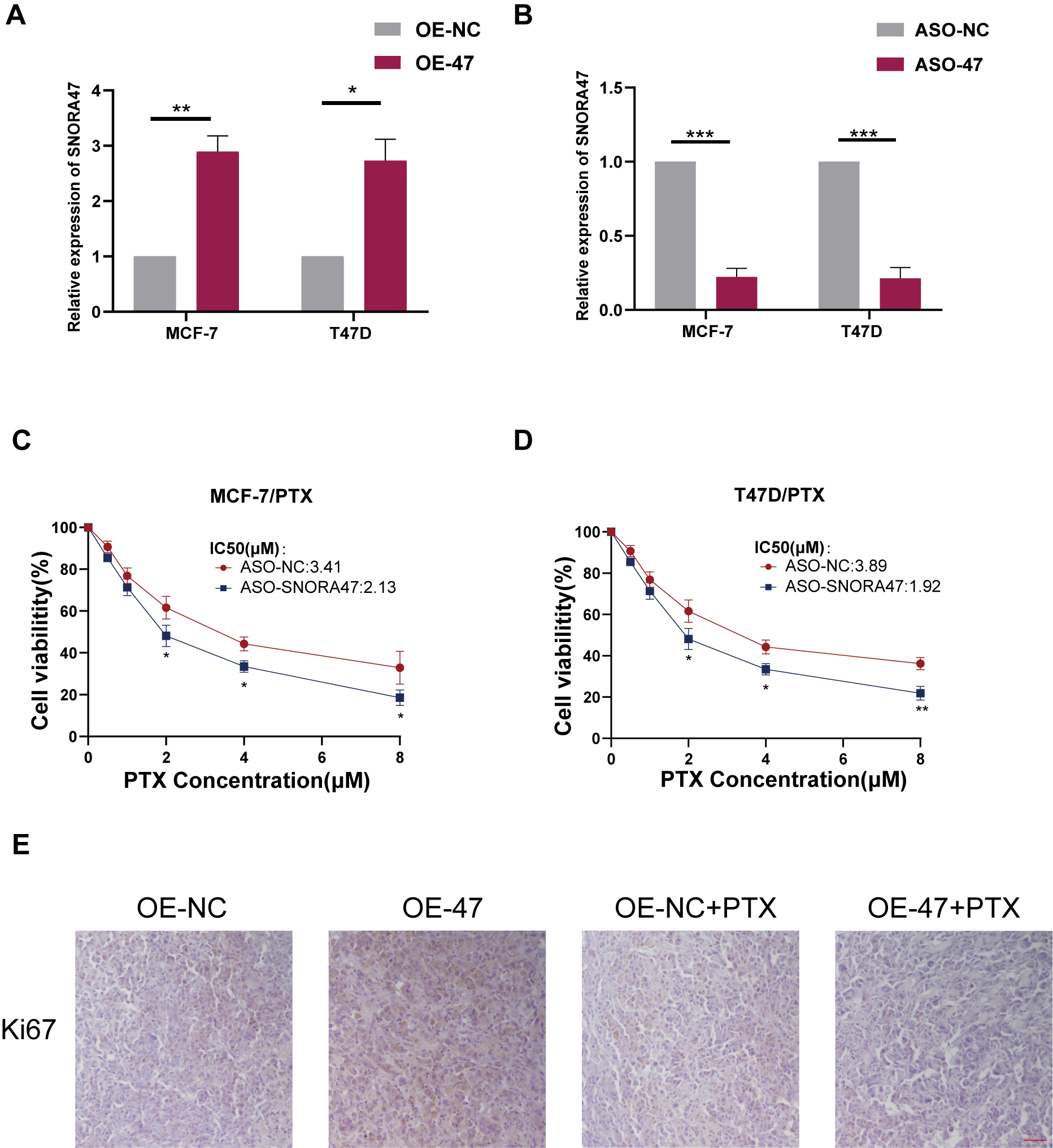

Supplement: Supplementary file 2 — Supplementary Material 2: Fig.S2.Silencing SNORA47 expression enhances drug sensitivity in breast cancer cells. A. qRT-PCR analysis of SNORA47 in MCF-7 and T47D cells after overexpression of SNORA47 (OE-47). B. qRT-PCR analysis of SNORA47 in MCF-7 and T47D cells after transfection with anti-SNORA47 ASOs (ASO-47). C-D. Cell viability and IC50 values of different concentrations of paclitaxel in MCF-7 and T47D cells after transfection with anti-SNORA47 ASOs. E. Ki67 protein levels were detected in tumor tissues with or without PTX treatment by IHC. Scale bar =50 m. All the results are displayed as the means SDs. *P < 0.05, **P <0.01, ***P < 0.001, ****P <0.0001; unpaired two-tailed Student’s t test. [file 10020_2025_1216_MOESM2_ESM.tif]

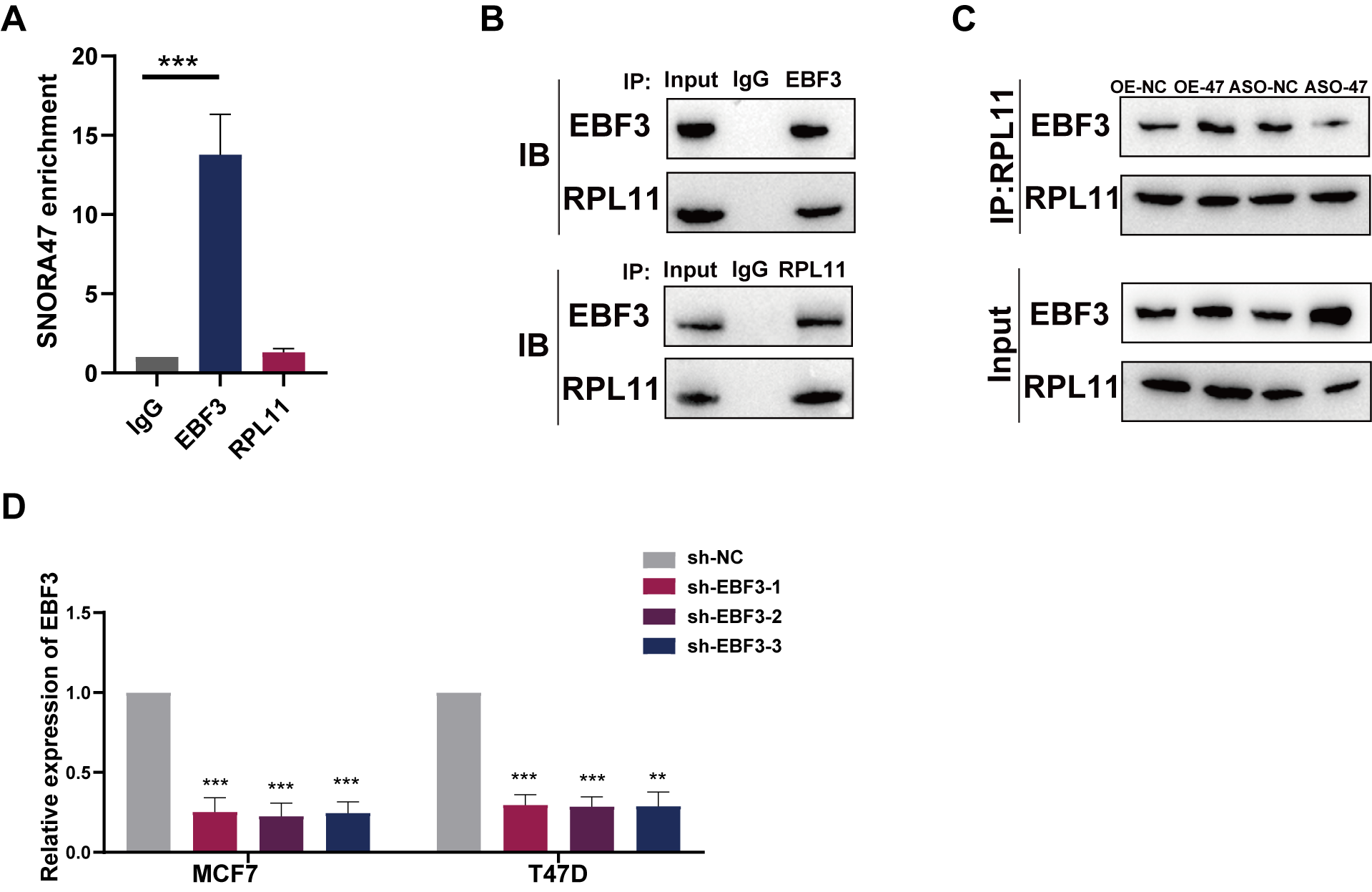

Supplement: Supplementary file 3 — Supplementary Material 3: Fig.S3. SNORA47 promotes interactions between EBF3 and RPL11.A. RNA immunoprecipitation (RIP) was performed using IgG, EBF3 and RPL11 antibodies with lysates from MCF-7 cells. Enrichment of SNORA47 was measured using qRT-PCR. B-C. Co-IP assay showing that EBF3 interacted with RPL11 (B) and changed (C) EBF3-RPL11 interactions after transfection with SNORA47 or anti-SNORA47 ASOs in T47D cells. D. qRT-PCR analyses of EBF3 in MCF-7 and T47D cells after EBF3 was knocked down. Each experiment was repeated independently at least three times. All the results are displayed as the means SDs. *P < 0.05, **P < 0.01, ***P < 0.001, ****P <0.0001; unpaired two-tailed Student’s t test. [file 10020_2025_1216_MOESM3_ESM.tif]

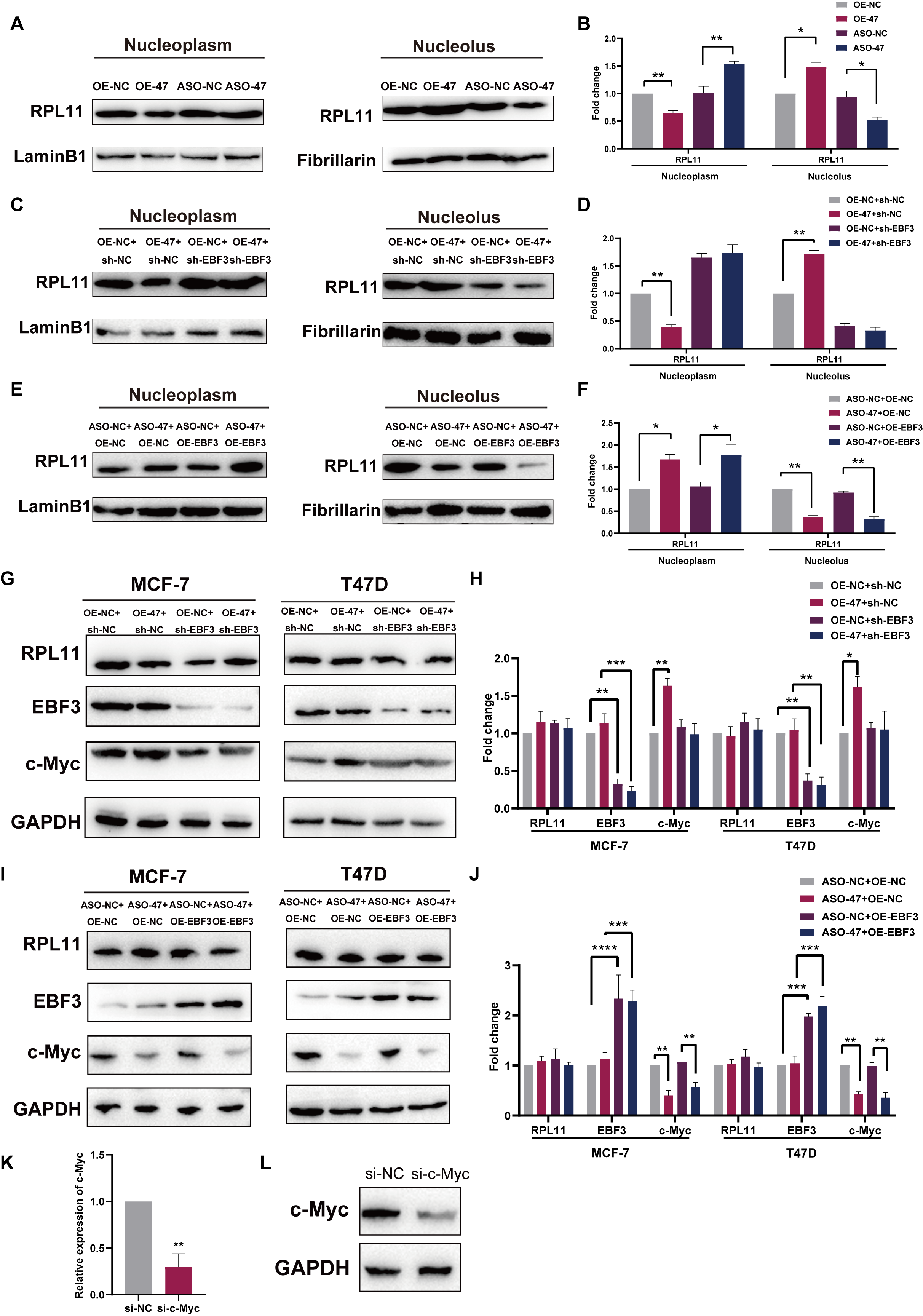

Supplement: Supplementary file 4 — Supplementary Material 4: Fig.S4. SNORA47 affects c-Myc expression via EBF3.A-F. Western blotting analysis of RPL11 in T47D cells after nucleolus-nucleoplasm separation. G-J. Western blotting analysis of RPL11, EBF3 and c-Myc in MCF-7 and T47D cells. K. qRT-PCR analyses of c-Myc in MCF-7 cells after c-Myc was knocked down. L. Western blotting analyses of c-Myc in MCF-7 cells after c-Myc was knocked down. Each experiment was repeated independently at least three times. All the results are displayed as the means SDs. *P < 0.05, **P <0.01, ***P < 0.001, ****P <0.0001; unpaired two-tailed Student’s t test. [file 10020_2025_1216_MOESM4_ESM.tif]
