# Supplementary material for: SNORA47 affects stemness and chemotherapy sensitivity via EBF3/RPL11/c-Myc axis in luminal A breast cancer
Source: Mol Med. 2025 Apr 22;31:150. doi: 10.1186/s10020-025-01216-3 (PMC12016144; doi:10.1186/s10020-025-01216-3)
Supplement: Supplementary file 5 — Supplementary Material 5: Table S1. The relationship of SNORA47 and NACT response [file 10020_2025_1216_MOESM5_ESM.docx]

**Table S1. The relationship of SNORA47 and NACT response**

| **Factors** | **Number** | **SNORA47 expression** | | ***P-value*** |
| --- | --- | --- | --- | --- |
|  |  | **Low** | **High** |  |
| **NACT clinical response** |  |  |  | 0.14 |
| **CB** | 17 | 11 | 6 |  |
| **Non CB** | 13 | 4 | 9 |  |
| **NACT pathological response** |  |  |  | 0.33 |
| **pCR** | 5 | 4 | 1 |  |
| **Non pCR** | 25 | 11 | 14 |  |
