# Supplementary material for: SNORA47 affects stemness and chemotherapy sensitivity via EBF3/RPL11/c-Myc axis in luminal A breast cancer
Source: Mol Med. 2025 Apr 22;31:150. doi: 10.1186/s10020-025-01216-3 (PMC12016144; doi:10.1186/s10020-025-01216-3)
Supplement: Supplementary file 6 — Supplementary Material 6: Table S2. Candidate proteins identified by RNA pull-down/LC-MS assays. [file 10020_2025_1216_MOESM6_ESM.docx]

**Table S2. Candidate proteins identified by RNA pull-down/LC-MS assays.**

| **Protein IDs** | **Protein names** | **Gene names** | **Fold of**  **change** |
| --- | --- | --- | --- |
| Q9NWT1 | p21-activated protein kinase-interacting protein 1 | PAK1IP1 | 5.43 |
| Q9NX58  Q9Y266  Q9H4W6  P22087  Q07020  Q15637  P62913  Q9UH1  Q99567  Q9UMS4  P78371  O75367  P46779  Q8NC51  Q9Y5B9  Q9H2D6  Q9BZI7  P46777  Q7KZF4 | Cell growth-regulating nucleolar protein  Nuclear migration protein nudC  Transcription factor COE3  Methyltransferase fibrillarin  60S ribosomal protein L18  Splicing factor 1  60S ribosomal protein L11  Poly(U)-binding-splicing factor PUF60  Nuclear pore complex protein Nup88  Pre-mRNA-processing factor 19  T-complex protein 1 subunit beta  Core histone macro-H2A.1  60S ribosomal protein L28  Plasminogen activator inhibitor 1 RNA-binding protein  FACT complex subunit SPT16  TRIO and F-actin-binding protein  Regulator of nonsense transcripts 3B  60S ribosomal protein L5  Staphylococcal nuclease domain-containing protein 1 | LYAR  NUDC  EBF3  FBL  RPL18  SF1  RPL11  PU60  NUP88  PRPF19  CCT2  H2AFY  RPL28  SERBP1  SUPT16H  TRIOBP  UPF3B  RPL5  SND1 | 4.32  4.12  3.76  3.45  3.01  2.67  2.31  2.11  1.98  1.87  1.72  1.69  1.60  1.57  1.57  1.31  1.26  1.22  1.20 |
